# (*E*)-2-(2-Nitro­prop-1-en­yl)furan

**DOI:** 10.1107/S1600536809030141

**Published:** 2009-07-31

**Authors:** Pedro Valerga, M. Carmen Puerta, Zenaida Rodríguez Negrín, Nilo Castañedo Cancio, Miguel Palma Lovillo

**Affiliations:** a Departamento de Ciencia de los Materiales e Ingeniería Metalúrgica, Facultad de Ciencias, Campus Universitario del Río San Pedro, Puerto Real 11510, Spain; b Centro de Bioactivos Químicos, Universidad Central Marta Abreu de Las, Villas, Cuba; c Departamento de Química Analítica, Facultad de Ciencias, Campus Universitario del Río San Pedro, Puerto Real 11510, Spain

## Abstract

Crystals of the title compound, C_7_H_7_NO_3_, under Mo Kα radiation sublime in less than 1h at room temperature. However, it was possible to collect data at 100K. It crystallized as the *E* isomer only. A double-bond conjugation in the furan ring is extended to the nitro­alkenyl group. Mol­ecular associations were realized in the crystal through N⋯π [3.545 (2) Å] inter­actions involving the furan ring and C—H⋯O hydrogen bonds.

## Related literature

For general background to (nitro-alken­yl)-furan compounds, see: Yan *et al.* (2008 ▶); Ono N. (2006 ▶); Vallejos *et al.* (2005 ▶); Negrín *et al.* (2003 ▶); Negrín *et al.* (2002 ▶), Estrada *et al.* (1999 ▶); Agafonov *et al.* (1991 ▶); Gruntfest *et al.* (1972 ▶). For related structures, see: Valerga *et al.* (2009 ▶); Martínez-Bescos *et al.* (2008 ▶); Novoa-de-Armas *et al.* (1997 ▶); Pomés *et al.* (1995 ▶). 
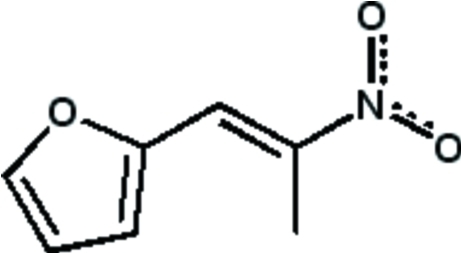


## Experimental

### 

#### Crystal data

C_7_H_7_NO_3_
*M* *_r_* = 153.14
Monoclinic, 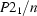

*a* = 7.1061 (14) Å
*b* = 9.4394 (19) Å
*c* = 10.743 (2) Å
β = 101.86 (3)°
*V* = 705.2 (3) Å^3^
*Z* = 4
Mo *K*α radiation
μ = 0.12 mm^−1^
*T* = 100 K
0.45 × 0.30 × 0.18 mm

#### Data collection

Bruker SMART APEX diffractometer
Absorption correction: multi-scan (*SADABS*; Sheldrick, 2004 ▶) *T*_min_ = 0.898, *T*_max_ = 1.000 (expected range = 0.880–0.980)
5648 measured reflections
1620 independent reflections
1497 reflections with *I* > 2σ(*I*)
*R*_int_ = 0.032

#### Refinement

*R*[*F* ^2^ > 2σ(*F* ^2^)] = 0.049
*wR*(*F* ^2^) = 0.130
*S* = 1.07
1620 reflections
102 parameters
H-atom parameters constrained
Δρ_max_ = 0.29 e Å^−3^
Δρ_min_ = −0.34 e Å^−3^

### 

Data collection: *SMART* (Bruker, 2001 ▶); cell refinement: *SAINT* (Bruker, 2001 ▶); data reduction: *SAINT*; program(s) used to solve structure: *SHELXTL* (Sheldrick, 2008 ▶); program(s) used to refine structure: *SHELXTL*; molecular graphics: *ORTEP-3* (Farrugia, 1997 ▶); software used to prepare material for publication: *SHELXTL*.

## Supplementary Material

Crystal structure: contains datablocks global, I. DOI: 10.1107/S1600536809030141/kp2230sup1.cif

Structure factors: contains datablocks I. DOI: 10.1107/S1600536809030141/kp2230Isup2.hkl

Additional supplementary materials:  crystallographic information; 3D view; checkCIF report

## Figures and Tables

**Table 1:** Hydrogen-bond geometry (Å, °)

*D*—H⋯*A*	*D*—H	H⋯*A*	*D*⋯*A*	*D*—H⋯*A*
C2—H2⋯O3^i^	0.95	2.47	3.3037 (19)	147
C5—H5⋯O3^i^	0.95	3.03	3.770 (2)	136
C4—H4⋯O2^ii^	0.95	2.65	3.2980 (19)	126
C4—H4⋯O3^ii^	0.95	2.58	3.516 (2)	170
C7—H7*C*⋯O2^iii^	0.98	2.70	3.310 (2)	121

Symmetry codes: (i) 
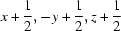
; (ii) 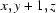; (iii) 
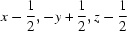
.

**Fig. 1.**
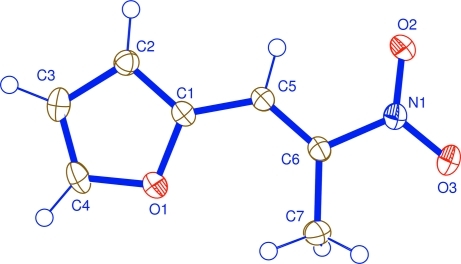
ORTEP representation of I with the atom labelling scheme and displacement ellipsoids at the 50% probability level.

**Fig. 2.**
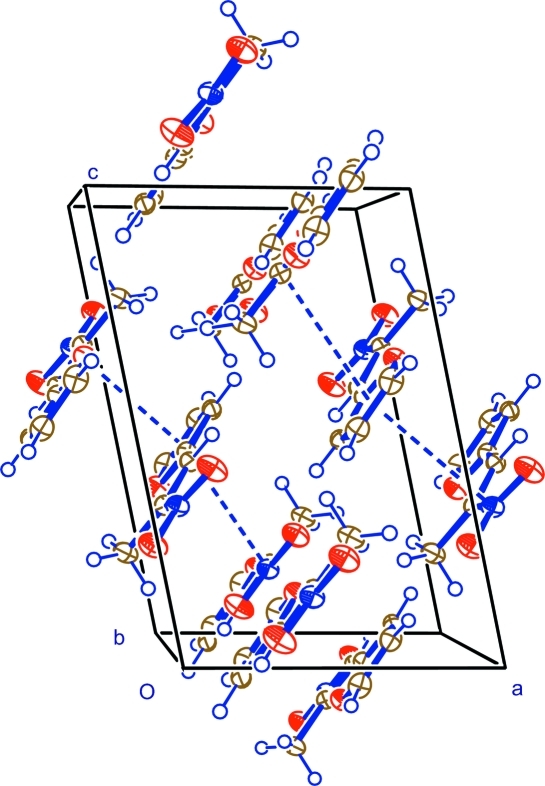
Packing diagram of E-2-(2-nitro-propen-1-yl)-furan.

## supplementary crystallographic information

### Comment

Some (nitro-alkenyl)-furan compounds show antibacterial and antifungal activities and were described and patented as drugs ingredients for veterinarian and agricultural purposes. We recently started the structural study of this group of furylnitroolefins. The title compound crystallized exclusively in its *E* configuration (Fig. 1). The conjugated system of double bonds in furan ring is extended to the alkenyl group being C2—C3 and C1—C5 bond lenghts 1.422 (2) and 1.431 (2) Å, significatively shorter than single C—C bonds. Distances N1—O2 = 1.232 (2) and N1—O3= 1.231 (2) indicate conjugation with a delocalized double bond. Alkenyl C5 and C6 *sp*^2^ carbons are coplanar with the furan ring as shown by an angle of 3.9 (1)° between ring plane and C5 C6 C7 N1 plane. Crystal packing shows N···π interactions involving the furan ring: N···Cg (1/2-x, -1/2+y,1/2-z) distance is 3.545 (2) Å (Fig. 2) and CH···O hydrogen bonds (Table 1).

### Experimental

2-(2-Nitro-propen-1-yl)-furan, also called UC-244, was obtained using the Knoevenagel's condensation method by reaction of furfural, an aromatic compound from acid hydrolysis of sugar cane residuals (straw, sawdust, etc.) and nitroethane in the presence of isobutylamine as a catalyst. To obtain a product with purity higher than 99% the method was optimized studying temperature, contact and reaction times as variables. The purification was achieved using activated coal and ethanol. The yellow crystals should be protected from the light and heating. ^1^H NMR (CDCl_3_) δ (ppm): 2.511 (3H, s, -C*H*_3_), 6.533 (1H, dd, ^2^*J* = 3.6 Hz and ^2^*J* = 1.6 Hz, -O-CH=C*H*-CH=), 6.781 (1H, d, ^2^*J* = 3.6Hz, -O-C*H*=CH-CH=), 7.599 (1H, d, ^2^*J* = 1.6 Hz, -O-CH=CH-C*H*=), 7.775 (1H, s, *H*C=CMe) ^13^C{^1^H} NMR (CDCl_3_) δ (ppm): 13.656 (-*C* H_3_), 112.688 (-O-CH=*C*H-CH=), 119.100 (-O-CH=CH-*C*H=), 120.346 (-*C*=C(Me)NO_2_), 144.097 (-C=*C*(Me)NO_2_), 146.104 (-O-*C*H=CH-CH=), 147.679 (*C*_ring_-CH-C=C(Me)NO_2_).

### Refinement

All H atoms were positioned geometrically and treated as riding (C—H = 0.99Å for methylene and C—H = 0.93Å otherwise). *U*_iso_(H) = 1.2 *U*_eq_(C) of the carrier atom. In the absence of any significant anomalous scatters, the Friedel pairs were merged before final refinements.

### Crystal data

**Table:**

C_7_H_7_NO_3_	*F*(000) = 320
*M**_r_* = 153.14	*D*_x_ = 1.442 Mg m^−^^3^
Monoclinic, *P*2_1_/*n*	Mo *K*α radiation, λ = 0.71073 Å
Hall symbol: -P 2yn	Cell parameters from 2619 reflections
*a* = 7.1061 (14) Å	θ = 2.9–27.6°
*b* = 9.4394 (19) Å	µ = 0.12 mm^−^^1^
*c* = 10.743 (2) Å	*T* = 100 K
β = 101.86 (3)°	Irregular, yellow
*V* = 705.2 (3) Å^3^	0.45 × 0.30 × 0.18 mm
*Z* = 4	

### Data collection

**Table:**

Bruker SMART APEX diffractometer	1620 independent reflections
Radiation source: fine-focus sealed tube	1497 reflections with *I* > 2σ(*I*)
graphite	*R*_int_ = 0.032
1700 ω scan frames, 0.3°, 10s	θ_max_ = 27.6°, θ_min_ = 2.9°
Absorption correction: multi-scan (*SADABS*; Sheldrick, 2004)	*h* = −9→8
*T*_min_ = 0.898, *T*_max_ = 1.000	*k* = −12→12
5648 measured reflections	*l* = −13→13

### Refinement

**Table:**

Refinement on *F*^2^	0 constraints
Least-squares matrix: full	H-atom parameters constrained
*R*[*F*^2^ > 2σ(*F*^2^)] = 0.049	*w* = 1/[σ^2^(*F*_o_^2^) + (0.069*P*)^2^ + 0.2975*P*] where *P* = (*F*_o_^2^ + 2*F*_c_^2^)/3
*wR*(*F*^2^) = 0.130	(Δ/σ)_max_ < 0.001
*S* = 1.07	Δρ_max_ = 0.29 e Å^−^^3^
1620 reflections	Δρ_min_ = −0.34 e Å^−^^3^
102 parameters	Extinction correction: *SHELXL*, Fc^*^=kFc[1+0.001xFc^2^λ^3^/sin(2θ)]^-1/4^
0 restraints	Extinction coefficient: 0.012 (3)

### Special details

**Table:**

Experimental. Refinement of F^2^ against unique set of reflections. The weighted *R*-factor *wR* and goodness of fit S are based on F^2^, conventional *R*-factors *R* are based on F, with F set to zero for negative F^2^. The threshold expression of F^2^ > 2sigma(F^2^) is used only for calculating *R*-factors(gt) *etc*. and is not relevant to the choice of reflections for refinement. *R*-factors based on F^2^ are statistically about twice as large as those based on F, and R– factors based on ALL data will be even larger.
Geometry. All e.s.d.'s (except the e.s.d. in the dihedral angle between two l.s. planes) are estimated using the full covariance matrix. The cell e.s.d.'s are taken into account individually in the estimation of e.s.d.'s in distances, angles and torsion angles; correlations between e.s.d.'s in cell parameters are only used when they are defined by crystal symmetry. An approximate (isotropic) treatment of cell e.s.d.'s is used for estimating e.s.d.'s involving l.s. planes.
Refinement. Refinement of *F*^2^ against unique set of reflections. The weighted *R*-factor *wR* and goodness of fit *S* are based on *F*^2^, conventional *R*-factors *R* are based on *F*, with *F* set to zero for negative *F*^2^. The threshold expression of *F*^2^ > σ(*F*^2^) is used only for calculating *R*-factors(gt) *etc*. and is not relevant to the choice of reflections for refinement. *R*-factors based on *F*^2^ are statistically about twice as large as those based on *F*, and *R*- factors based on ALL data will be even larger.

### Fractional atomic coordinates and isotropic or equivalent isotropic displacement parameters (Å^2^)

**Table:**

	*x*	*y*	*z*	*U*_iso_*/*U*_eq_	
O1	0.08533 (15)	0.64923 (11)	0.35499 (10)	0.0257 (3)	
O2	0.22698 (17)	0.12565 (12)	0.41245 (11)	0.0328 (3)	
O3	−0.00888 (16)	0.10699 (11)	0.25033 (11)	0.0311 (3)	
N1	0.09873 (17)	0.17838 (13)	0.33088 (11)	0.0227 (3)	
C1	0.19682 (19)	0.55649 (15)	0.43728 (13)	0.0200 (3)	
C2	0.31539 (19)	0.62992 (15)	0.53073 (13)	0.0211 (3)	
H2	0.4068	0.5911	0.5993	0.025*	
C3	0.2760 (2)	0.77609 (16)	0.50590 (15)	0.0272 (4)	
H3	0.3360	0.8539	0.5545	0.033*	
C4	0.1374 (2)	0.78256 (15)	0.40026 (16)	0.0278 (4)	
H4	0.0828	0.8679	0.3620	0.033*	
C5	0.18569 (19)	0.40627 (15)	0.41909 (12)	0.0194 (3)	
H5	0.2709	0.3514	0.4802	0.023*	
C6	0.07004 (19)	0.33308 (14)	0.32658 (12)	0.0197 (3)	
C7	−0.0833 (2)	0.38425 (16)	0.22071 (14)	0.0266 (3)	
H7A	−0.1245	0.4791	0.2409	0.040*	
H7B	−0.1932	0.3193	0.2092	0.040*	
H7C	−0.0336	0.3881	0.1422	0.040*	

### Atomic displacement parameters (Å^2^)

**Table:**

	*U*^11^	*U*^22^	*U*^33^	*U*^12^	*U*^13^	*U*^23^
O1	0.0287 (6)	0.0186 (5)	0.0277 (5)	0.0010 (4)	0.0008 (4)	0.0023 (4)
O2	0.0399 (7)	0.0183 (5)	0.0339 (6)	0.0058 (5)	−0.0073 (5)	0.0003 (4)
O3	0.0372 (7)	0.0217 (6)	0.0304 (6)	−0.0050 (4)	−0.0023 (5)	−0.0073 (4)
N1	0.0263 (6)	0.0186 (6)	0.0223 (6)	−0.0007 (5)	0.0028 (5)	−0.0024 (5)
C1	0.0210 (7)	0.0172 (7)	0.0222 (7)	0.0015 (5)	0.0051 (5)	0.0019 (5)
C2	0.0197 (7)	0.0216 (7)	0.0217 (7)	−0.0002 (5)	0.0038 (5)	−0.0009 (5)
C3	0.0296 (8)	0.0210 (7)	0.0324 (8)	−0.0057 (6)	0.0100 (6)	−0.0069 (6)
C4	0.0321 (8)	0.0145 (7)	0.0383 (8)	0.0012 (6)	0.0105 (6)	0.0031 (6)
C5	0.0199 (7)	0.0173 (7)	0.0206 (6)	0.0013 (5)	0.0034 (5)	0.0010 (5)
C6	0.0217 (7)	0.0164 (6)	0.0211 (7)	0.0014 (5)	0.0047 (5)	0.0005 (5)
C7	0.0292 (8)	0.0254 (7)	0.0224 (7)	0.0037 (6)	−0.0014 (6)	−0.0020 (6)

### Geometric parameters (Å, °)

**Table:**

O1—C4	1.3724 (18)	C3—C4	1.342 (2)
O1—C1	1.3739 (17)	C3—H3	0.9500
O2—N1	1.2320 (16)	C4—H4	0.9500
O3—N1	1.2311 (16)	C5—C6	1.3434 (19)
N1—C6	1.4739 (18)	C5—H5	0.9500
C1—C2	1.3602 (19)	C6—C7	1.4850 (19)
C1—C5	1.431 (2)	C7—H7A	0.9800
C2—C3	1.422 (2)	C7—H7B	0.9800
C2—H2	0.9500	C7—H7C	0.9800
			
C4—O1—C1	106.17 (12)	C3—C4—H4	124.6
O3—N1—O2	122.73 (12)	O1—C4—H4	124.6
O3—N1—C6	117.28 (11)	C6—C5—C1	128.22 (13)
O2—N1—C6	119.99 (11)	C6—C5—H5	115.9
C2—C1—O1	109.76 (13)	C1—C5—H5	115.9
C2—C1—C5	127.88 (13)	C5—C6—N1	115.26 (12)
O1—C1—C5	122.34 (12)	C5—C6—C7	129.82 (13)
C1—C2—C3	106.73 (13)	N1—C6—C7	114.92 (12)
C1—C2—H2	126.6	C6—C7—H7A	109.5
C3—C2—H2	126.6	C6—C7—H7B	109.5
C4—C3—C2	106.53 (13)	H7A—C7—H7B	109.5
C4—C3—H3	126.7	C6—C7—H7C	109.5
C2—C3—H3	126.7	H7A—C7—H7C	109.5
C3—C4—O1	110.81 (13)	H7B—C7—H7C	109.5
			
C4—O1—C1—C2	−0.25 (15)	O1—C1—C5—C6	−1.4 (2)
C4—O1—C1—C5	−178.62 (12)	C1—C5—C6—N1	177.53 (12)
O1—C1—C2—C3	0.10 (15)	C1—C5—C6—C7	−2.8 (2)
C5—C1—C2—C3	178.36 (13)	O3—N1—C6—C5	177.57 (12)
C1—C2—C3—C4	0.08 (16)	O2—N1—C6—C5	−2.79 (19)
C2—C3—C4—O1	−0.24 (17)	O3—N1—C6—C7	−2.15 (18)
C1—O1—C4—C3	0.31 (16)	O2—N1—C6—C7	177.49 (12)
C2—C1—C5—C6	−179.46 (13)		

### Hydrogen-bond geometry (Å, °)

**Table:**

*D*—H···*A*	*D*—H	H···*A*	*D*···*A*	*D*—H···*A*
C2—H2···O3^i^	0.95	2.47	3.3037 (19)	147
C5—H5···O3^i^	0.95	3.03	3.770 (2)	136
C4—H4···O2^ii^	0.95	2.65	3.2980 (19)	126
C4—H4···O3^ii^	0.95	2.58	3.516 (2)	170
C7—H7C···O2^iii^	0.98	2.70	3.310 (2)	121

Symmetry codes: (i) *x*+1/2, −*y*+1/2, *z*+1/2; (ii) *x*, *y*+1, *z*; (iii) *x*−1/2, −*y*+1/2, *z*−1/2.
